# Gut Dysbiosis and *Clostridioides difficile* Infection in Neonates and Adults

**DOI:** 10.3389/fmicb.2021.651081

**Published:** 2022-01-20

**Authors:** Iulia-Magdalena Vasilescu, Mariana-Carmen Chifiriuc, Gratiela Gradisteanu Pircalabioru, Roxana Filip, Alexandra Bolocan, Veronica Lazăr, Lia-Mara Diţu, Coralia Bleotu

**Affiliations:** ^1^Department of Microbiology, Faculty of Biology, University of Bucharest, Bucharest, Romania; ^2^INBI “Prof. Dr. Matei Balş” – National Institute for Infectious Diseases, Bucharest, Romania; ^3^Research Institute of the University of Bucharest, Bucharest, Romania; ^4^Academy of Romanian Scientists, Bucharest, Romania; ^5^The Romanian Academy, Bucharest, Romania; ^6^Faculty of Medicine and Biological Sciences, Stefan cel Mare University of Suceava, Suceava, Romania; ^7^Regional County Emergency Hospital, Suceava, Romania; ^8^Department of General Surgery, University Emergency Hospital, Carol Davila University of Medicine and Pharmacy, Bucharest, Romania; ^9^Ştefan S. Nicolau Institute of Virology, Romanian Academy, Bucharest, Romania

**Keywords:** *Clostridium difficile* infection, gut microbiota, dysbiosis, biotics, fecal microbiota transplantation

## Abstract

In this review, we focus on gut microbiota profiles in infants and adults colonized (CDC) or infected (CDI) with *Clostridioides difficile*. After a short update on CDI epidemiology and pathology, we present the gut dysbiosis profiles associated with CDI in adults and infants, as well as the role of dysbiosis in *C. difficile* spores germination and multiplication. Both molecular and culturomic studies agree on a significant decrease of gut microbiota diversity and resilience in CDI, depletion of *Firmicutes, Bacteroidetes*, and *Actinobacteria* phyla and a high abundance of *Proteobacteria*, associated with low butyrogenic and high lactic acid-bacteria levels. In symptomatic cases, microbiota deviations are associated with high levels of inflammatory markers, such as calprotectin. In infants, colonization with *Bifidobacteria* that trigger a local anti-inflammatory response and abundance of *Ruminococcus*, together with lack of receptors for clostridial toxins and immunological factors (e.g., *C. difficile* toxins neutralizing antibodies) might explain the lack of clinical symptoms. Gut dysbiosis amelioration through administration of “biotics” or non-toxigenic *C. difficile* preparations and fecal microbiota transplantation proved to be very useful for the management of CDI.

## Introduction

*Clostridioides* (formerly *Clostridium*) *difficile* is a Gram-positive, obligate anaerobe, spore-forming bacteria, harboring a plethora of surface and secreted proteins responsible for the colonic colonization and subsequent inflammation characteristic for *C. difficile* infection (CDI), among which the most important are the clostridial toxins: toxin A (TcdA) and toxin B (TcdB), and in some bacterial strains, the binary toxin CDT (Smits et al., 2016). Clinical symptoms range from mild diarrhea to fulminant colitis, known as pseudomembranous colitis, with its complications – toxic megacolon and large bowel perforation (McDonald et al., 2018; Dieterle et al., 2019). *C. difficile* is the number one causative agent of nosocomial post-antibiotic colitis, associated with high morbidity and mortality (Ghose, 2013). In the last decade, both frequency and severity of CDI have increased, largely due to the emergence of a hypervirulent strain called NAP1 (North American pulsed-field gel electrophoresis type 1 strain) (Depestel and Aronoff, 2013). Moreover, in the last two decades, there has been a significant increase in the incidence of CDI in previously considered low-risk population categories, including community-associated-*Clostridioides difficile* infections (CA-CDI), with more than 30% of cases not showing typical CDI risk factors, such as antibiotic treatment or recent hospitalization (Wilcox et al., 2008; Hensgens et al., 2011). Although CDI is the preserve of the elderly population, it can also affect other age segments. In a retrospective survey performed by U.S. National Hospital Discharge Surveys from 2001 to 2010 revealed that CDI incidence was highest among elderly adults (11.6 CDI discharges/1,000 total discharges), followed by adults (3.5 CDI discharges/1,000 total discharges) and pediatrics (<12 years) (1.2 CDI discharges/1,000 total discharges). The mortality rates attributable to CDI in the elderly were significantly higher (8.8%) compared to adults (3.1%) and pediatrics (1.4%) (Pechal et al., 2016). Although there is not much information available about the epidemiology of infection in infants, however, the carriage rate of non-toxigenic *C. difficile* is very high in newborns, suggesting the commensal status of this bacterium in this population segment (Sammons et al., 2013; Borali and De Giacomo, 2016). For this reason, *C. difficile* is not considered an enteric pathogen in infants and children affected by bloody diarrhea and younger than 12 months (Cama et al., 2019). However, asymptomatic infants could be also infected by toxigenic adult infectious strains both after hospitalization and even in the community, thus, constituting a reservoir for toxigenic strains (Rousseau et al., 2011; Ferraris et al., 2020).

This review aims to present some particular aspects of gut microbiota in CDI infants and adults, taking into account that the pathophysiology of this disease suggests that the clinical manifestations occur in cases of an imbalance of the intestinal microbiota, known as dysbiosis. In this purpose, studies performing culture-dependent (*C. difficile* cultivation) and independent (16S rRNA and metagenomics) have been analyzed.

Some of the underlying causes of intestinal dysbiosis are antibiotic treatments (Kukla et al., 2020), advanced age (over 65 years), hospitalization (particularly in patients sharing the hospital room with an infected patient, in intensive care units, during prolonged hospitalization), nursing home stay, severe associated diseases, immunological suppression, gastric acidity suppression by proton pump inhibitors or histamine_2_-receptor antagonists and prolonged use of elemental diet in the context of enteral nutrition, inflammatory bowel diseases, gastrointestinal surgery (in particular colectomy, small-bowel resection, and gastric resection were associated with the highest risk while patients undergoing cholecystectomy and appendectomy had the lowest risk), all of these circumstances being associated with characteristic changes in the configuration of the gut microbiota and with an increased CDI risk (O’Keefe, 2010; Fashner et al., 2011; Nitzan et al., 2013; Rodriguez et al., 2016; Sartelli et al., 2019; Avni et al., 2020). A strong argument regarding the impact of dysbiosis on CDI risk is offered by animal studies proving that correcting dysbiosis by administration of different substances such as phytophenolic compounds or carvacrol has been shown to decrease susceptibility to CDI. The gut dysbiosis of 6-week-old C57BL/6 mice was induced by the oral administration of an antibiotic cocktail in water simultaneously with the intra-peritoneal injection of clindamycin. The mice were infected with 10^5^ CFU/ml of hypervirulent *C. difficile* ATCC 1870 spores. Carvacrol supplementation significantly reduced the incidence of diarrhea and improved mice’s clinical and diarrhea scores. Microbiome analysis revealed that carvacrol increased the abundance of *Bacteroidetes* and *Firmicutes*. An increased abundance of *Lactobacillaceae* and *Lachnospiraceae* was noticed among the beneficial taxa in carvacrol treated mice. Also, carvacrol decreased the proportion of pro-inflammatory microbiota, such as *Proteobacteria* (i.e., *Enterobacteriaceae*) and *Verrucomicrobia*, without significantly affecting the gut microbiome diversity compared to the control (Mooyottu et al., 2017).

Five clinical features (potential risk factors) predict dysbiosis in CDI patients: antibiotic use within the previous 3 weeks, immunosuppression, multimorbidity, recent/multiple hospitalization, and prior CDI (Battaglioli et al., 2018). In individuals whose normal intestinal microbiota has been disrupted, ingested *C. difficile* spores germinate in the presence of bile salts in the small intestine and colonize the colon epithelial cells, releasing the inflammatory enterotoxins, which are primarily and largely responsible for the colonic inflammation in *C. difficile* diseases, inducing cytoskeletal changes, disruption of tight junctions, and induction of inflammatory cytokine production (Shen, 2012; Winston and Theriot, 2016). The *C. difficile* spores are released by the patient facilitating CDI transmission to susceptible hosts (Czepiel et al., 2019).

## *Clostridioides difficile* Infection and Dysbiosis in Adults

The intestinal environment represents a complex network of bacterial cells and metabolic products and/or other unknown substances derived from their own structures or metabolisms, which are in close and continuous interaction, both with each other, as well as with the human intestinal cells and the host’s immune system (Lazar et al., 2018, 2019).

The characterization of the baseline healthy microbiota and differences that are associated with various diseases has been possible with the contribution of large-scale projects, such as Meta-HIT and the Human Microbiome Project (HMP), using different omics technologies (Qin et al., 2010; Human Microbiome Project, 2012).

During early development, the gut microbiota undergoes subsequent changes until a stable adult state is reached. The adult microbiota has three basic characteristics: diversity (a high microbiota diversity defined by high species richness and high functional diversity being generally associated with the health condition), resilience (the property of the gut microbiota to resist to an impact and to recover and to baseline after the disturbance cessation; the capacity of a microbial community to reach a stable state in response to chemical, physical or biological perturbations of different intensities is achieved through genetically diverse resident clonal populations and population-level dynamics) and long-term stability of high taxonomic level components (Levine and d’Antonio, 1999; Lozupone et al., 2012; McBurney et al., 2019; Priya and Blekhman, 2019; Dogra et al., 2020).

Regarding the diversity, human microbiota displays a remarkable heterogeneity within and between individuals, the results of the culture-independent studies leading to the generally accepted idea that we rather share a functional core microbiome, than a core microbiota (Lozupone et al., 2012). The >1,000 estimated species-level phylotypes are belonging to few microbial phyla, which are *Firmicutes, Bacteroidetes, Actinobacteria, Proteobacteria, Fusobacteria*, and *Verrucomicrobia*; among these, the two *Firmicutes* and *Bacteroidetes* phyla are representing 90% of gut microbiota (Magne et al., 2020). *Firmicutes* comprises more than 200 different genera, such as *Lactobacillus, Bacillus, Clostridium, Enterococcus*, and *Ruminococcus* (Kachrimanidou and Tsintarakis, 2020). *Clostridium* genera represent 95% of the *Firmicutes*, while *Bacteroidetes* include as predominant genera *Bacteroidetes* and *Prevotella* (Rinninella et al., 2019). *Actinobacteria* are proportionally less abundant and mainly represented by *Bifidobacterium* species (Rinninella et al., 2019). From the mentioned components, *Ruminococcus* and *Bifidobacterium* have been reported to exhibit protective roles against CDI (Seekatz and Young, 2014; Kho and Lal, 2018).

The gut microbiota community shows resilience to different perturbations, such as those induced by different diets, antibiotic administration, invasion by new species (called colonization resistance) (Folke et al., 2004). Under the impact of a certain disturbances, such as antibiotic administration, microbiota enters an unstable state that progresses to a new stable state. When the latter is highly similar to the pre-disturbance state, this indicates a complete recovery. However, sometimes the post-disturbance stable state is distinct and this unfortunately can be both abnormal and resilient, as a response to the perturbation persistence (e.g., poor diet, antibiotic treatments etc) (Dupont, 2011). An example of gut microbiota resilience is the success of bacteriotherapy or microbiota transplantation in treating recurrent CDI. In this case, the gut microbiota switches from an initial dysbiosis state, favoring the CDI (e.g., increased abundance of *Veillonella* and *Streptococcus*) to a baseline state, in which the taxa from the healthy donor (e.g., *Bacteroidetes*) persisted 1 month after transplantation (Gough et al., 2011). A high diversity (high species richness, α-diversity) and host immune effectors, e.g., nucleotide-binding oligomerization domain (Nod2), an intracellular innate immune sensor involved in the anti-infectious host defense, were linked to gut microbiota resilience (Tap et al., 2015; Raymond et al., 2016; Goethel et al., 2019). A low microbiota diversity was correlated with recurrent CDI, but with unknown effects on microbiota resilience (Chang et al., 2008; Khoruts et al., 2010). However, a comparable phylum-level diversity was observed in individuals with initial CDI and healthy controls, while in case of recurrent CDI the phylum-level diversity switched to different highly divergent profiles very different from the healthy state other (Chang et al., 2008).

The stability of gut microbiota is affected by different factors, including genetic factors, early-life events, travel, dietary changes, weight loss or gain, diarrheal disease, antibiotics, immunosuppressants, premeditated interventions to influence the microbiota by administration of prebiotics, probiotics, postbiotics and symbiotics, as well as fecal transplantation (Faith et al., 2013; Britton and Young, 2014).

### Dysbiosis Profiles in *Clostridioides difficile* Infection Patients

In this section, we will present some of the culture-dependent and independent studies that have been performed to identify the dysbiosis profiles (Table 1) and specific microbial derived biomarkers in patients prone to CDI. A metagenomic and culturomic analysis of gut microbiota dysbiosis during CDI has shown a significant depletion of *Bacteroidetes* in *C. difficile* patients compared with the control group (Amrane et al., 2019). Diversity was significantly higher in the control group. *Proteobacteria* were more common in the CDI group. *Firmicutes* and *Actinobacteria* were less common in the CDI group (Lozupone et al., 2012). *Firmicutes* are involved in butyrate, and other short chain fatty acids (SCFA) production, these molecules playing a role in gut homeostasis and inhibition of *C. difficile* germination (Kachrimanidou and Tsintarakis, 2020) and *Bacteroidetes* are involved in carbohydrates digestion, producing substrates for colonocytes (den Besten et al., 2013). Depletion of these two major phyla of gut microbiota was detected in the *C. difficile* group. The bacterial families conferring resistance to CDI are *Bacteroidaceae, Bifidobacteriaceae*, and *Lachnospiraceae* (Antharam et al., 2013). Studies in animals with CDI, revealed a high abundance of *Proteobacteria* (especially *Enterobacteriaceae*) and a numerical decrease of *Lachnospiraceae* (*Firmicutes*) in diseased animals (Reeves et al., 2011). *Lachnospiraceae* strains have been shown to be able to partially restore colonization resistance, the mice inoculated with such strains showing decreased *C. difficile* colonization, lower levels of cytotoxins and lower clinical signs of severe infection (Reeves et al., 2012). An increased relative abundance of *Enterococcus, Lactobacillus, Escherichia coli*, *Enterobacter, Bacteroides*, *Parabacteroides, Akkermansia muciniphila*, and decreased *Faecalibacterium, Roseburia, Blautia, Prevotella, Megamonas*, *Streptococcus*, and *Bacteroides* levels were evidenced in the gut microbiota of CDI patients (Rea et al., 2012). The bacteria found only in the control group, which may have a role against *C. difficile*, were *Bacteroides ovatus, Bacteroides vulgatus* and *Oscillibacter massiliensis* (Lozupone et al., 2012). Only three bacteria with a potential role against *C. difficile* were detected both by culturomics and metagenomics, namely *Bifidobacterium adolescentis*, *Bifidobacterium longum* and *Bacteroides ovatus* (Lozupone et al., 2012). Overrepresentation of *Akkermansia* may be a predictive marker for the development of nosocomial diarrhea, with a worsened CDI prognosis (Hernandez et al., 2018; Vakili et al., 2020).

**TABLE 1:** Gut microbiota dysbiosis associated with *Clostridium difficile* infection in adults and infants (proposed microbiota-derived biomarkers for CDI dysbiosis are presented in bold).

Effect	Taxonomic level	Representatives	Mechanism	References
**Adults**				
Depletion	Gut microbiota	Cultivable/non-cultivable microbiota	Disrupted microbiota; decreased richness and diversity	Amrane et al., 2019
	Phylum	*Firmicutes*	Butyrate and short chain fatty acid production; role in gut homeostasis and inhibition of *C. difficile* germination	Antharam et al., 2013; den Besten et al., 2013; Abt et al., 2016
		*Bacteroidetes*	Carbohydrate digestion, producing substrates for colonocytes	den Besten et al., 2013; Zhang et al., 2015
		*Actinobacteria*		Amrane et al., 2019
	Families	*Bacteroidaceae*		Lozupone et al., 2012
		*Bifidobacteriaceae*		Lozupone et al., 2012
		*Lachnospiraceae*	Colonization resistance	Reeves et al., 2011, 2012; Antharam et al., 2013; Perez-Cobas et al., 2015
		*Clostridiales*	*C. difficile* spores germination inhibition and colonization	
		*Ruminococcaceae*		Franzosa et al., 2019; Lo Presti et al., 2019; Wilson et al., 2019; Berkell et al., 2021
	Genera and species	*Faecalibacterium, Roseburia, Blautia, Dorea*, *Prevotella, Megamonas*, *Subdoligranulum, Anaerostipes, Pseudobutyrivibrio, Streptococcus, Ezakiella, Odoribacter, Bacteroides sp.*, ***Alistipes***, ***B. ovatus***, *B. vulgatus*, ***Bifidobacterium adolescentis*, *B. longum***, *Oscillibacter massiliensis, Clostridium scindens*	Decrease of luminal pH by butyrogenic and acetogenic bacteria, stimulation of mucin and antimicrobial peptides production, maintaining decreased permeability Primary bile acids conversion Production of lantibiotics (nisin O)	Guilloteau et al., 2010; Lozupone et al., 2012; Antharam et al., 2013; Hamilton et al., 2013; Solomon, 2013; Gupta et al., 2016; Milani et al., 2016; Theriot et al., 2016; Winston and Theriot, 2016; Hatziioanou et al., 2017; Vakili et al., 2020
Increase	Phylum	*Proteobacteria*		Rodriguez et al., 2020
	Families	***Enterobacteriaceae***	Increased intestinal permeability	Collins and Auchtung, 2017
	Genera/species	*Finegoldia, Enterococcus, Lactobacillus, Fusobacterium, Mycobacterium, Enterobacter, Bacteroides*, *Parabacteroides, Escherichia coli*, ***Akkermansia muciniphila***	Lactic acid bacteria	Reeves et al., 2011, 2012; Na and Kelly, 2011; Rea et al., 2012; Rajilic-Stojanovic et al., 2013; Gevers et al., 2014; Milani et al., 2016; Ross et al., 2016
**Infants**				
Increase	Genera/species	*Staphylococcus aureus, Enterococcus*, *Escherichia coli, Shigella spp., Citrobacter spp., Klebsiella spp.*	Triggering a pro-inflammatory response	Heida et al., 2016
Decrease	Phylum	*Bacteroidetes, Firmicutes*		Dobbler et al., 2017
		*Bifidobacteria*	Upregulation of IL-10 production	Huurre et al., 2008
	Genera/species	*Ruminococcus*		Morelli, 2008

### Dysbiosis-Associated Biochemical Features in *Clostridioides difficile* Infection Patients

The gut dysbiosis also results in biochemical and immunological disruptions like decreased short chain fatty acids (SCFAs) levels, the abundance of primary bile acids, high availability of carbohydrates, suppression of immunological mechanisms and absence of competitors, all resulting in increased colonization capacity, favoring germination and growth of *C. difficile* (Ridlon et al., 2014; Carding et al., 2015; Goh and Klaenhammer, 2015; Valdes et al., 2018; Hills et al., 2019; Toor et al., 2019). Nosocomial diarrheal syndromes, including CDI, were associated not only with decreased bacterial diversity, *Firmicutes* paucity, low numbers of *Ruminococcaceae, Lachnospiraceae* but also with low levels of butyrogenic (e.g., *Roseburia, Faecalibacterium, Subdoligranulum, Anaerostipes*, and *Pseudobutyrivibrio*) and acetogenic (e.g., *Blautia* and *Dorea*) genera and with high levels of lactic acid bacteria (e.g., *Enterococcus* sp.) (Antharam et al., 2013). A decrease in butyrate-producing bacteria and an increase in lactic acid-producing bacteria were associated with increased CDI risk (Vakili et al., 2020). The role of SCFAs depletion in facilitating *C. difficile* infection is not yet elucidated, yet however, SCFAs could act by reducing the luminal pH (unfavorable for *C. difficile*) and stimulating the defensive barrier by production of mucin and antimicrobial peptides (defensins and cathelicidins, secreted by specialized cells, Paneth cells and leukocytes in the intestinal crypts) (Guilloteau et al., 2010; Solomon, 2013; Gupta et al., 2016). Among SCFAs, butyrate seems to have no effect on *C. difficile* colonization and toxin production, but it can protect the intestinal epithelium from the damage caused by *C. difficile* toxins by stabilizing hypoxia-inducible factor-1 (HIF-1) and increasing tight junctions, and thus decreasing intestinal epithelial permeability, inhibiting intestinal inflammation and bacterial translocation. The addition of butyrate to the drinking water of mice, administration of a pro-drug of butyrate, tributyrin, or of an inulin-rich diet (inulin can be fermented by gut commensal bacteria, which generate short-chain fatty acids, mainly acetate, propionate, and butyrate) resulted in the protection of mice against CDI (Fachi et al., 2019; Song et al., 2020).

*C. difficile* spore germination is regulated by the detection of bile salt and amino acid cogerminants by pseudoproteases CspC and CspA, respectively (Weingarden et al., 2014; Lawler et al., 2020). Although some cholate derivatives and the amino acid glycine could act as co-germinant factors, deoxycholate prevents vegetative growth (Sorg and Sonenshein, 2008), while chenodeoxycholate inhibits taurocholate-mediated germination (Sorg and Sonenshein, 2010). Commensal members of *Clostridiales* present in the gut contribute to the creation of an inappropriate environment for *C. difficile* germination and colonization by modulating the production of cogerminants (Perez-Cobas et al., 2015). For example, *Clostridium scindens*, a bile acid 7α-dehydroxylating intestinal strain, is associated with resistance to *C. difficile* infection, and, upon administration, it enhances resistance to infection in association with a secondary bile acid (Buffie et al., 2015). Depleting specific gut microbes responsible for converting primary bile acids into secondary antimicrobial bile acids could be associated with increased risk of CDI (Theriot et al., 2016; Winston and Theriot, 2016).

*Clostridium* species are among the best-described users of free amino acids as energy sources. Amino acids regulate *in vitro* toxin production and support colonization of *C. difficile* in antibiotic-treated mice. Dysbiotic microbial communities showed significantly decreased expression of multiple genes related to amino acid uptake and metabolism, resulting in increased concentrations of 12 amino acids, with proline showing significant differences when compared to healthy mice microbiota. The ability to utilize proline provides a competitive advantage to *C. difficile* in germ-free mice transplanted with healthy-like and dysbiotic human stool consortia. Fecal microbiota transplant reduced free proline and decreased CDI susceptibility in dysbiotic mice (Mooyottu et al., 2017).

Recent studies have found increased indole levels (tryptophan metabolite involved in microbial growth, virulence induction, acid resistance, biofilm development) in the intestinal lumen of CDI patients, suggesting that *C. difficile*, which cannot produce this metabolite itself, would stimulate the production of indole by other bacteria to stop the growth and the development of indole-sensitive strains, including protective gut microbiota representatives, thus ensuring an intestinal environment conducive to its survival (Darkoh et al., 2019).

A recent study evaluated the relationship between the composition of the intestinal microbiota and level of fecal calprotectin in *C. difficile* asymptomatic and symptomatic patients. The asymptomatic patients have shown a modified microbiota, comparatively with the non-colonized patients, harboring significantly lower levels of *Ruminococcaceae, Bilophila*, *Blautia, Faecalibacterium, Ruminococcus*, and *Sutterella*, and higher levels of *Enterobacteriaceae*. In symptomatic patients the main devations of gut microbiota were represented by higher levels of *Bacteroides* and lower levels of *Blautia*, *Phascolarctobacterium, Prevotella*, and *Succinivibrio*. These gut microbiota changes in symptomatic patients were accompanied by significantly higher levels of fecal calprotectin, comparatively with asymptomatic patients and controls. These data suggest that association of microbiota and inflammatory markers could be used to differentiate *C. difficile* colonization (CDC) from CDI (Han et al., 2020).

Knowledge regarding the gut microbiota in *C. difficile* colonized patients may elucidate the mechanisms that allow for colonization whilst protecting against infection (Crobach et al., 2020). To this end, a recent study by Crobach et al. (2020) analyzed the bacterial signatures associated with resistance and susceptibility to CDC and CDI. Both CDC and CDI were associated with decreased gut microbial diversity and differences in the relative abundance of taxa such as *Lachnospiraceae, Ruminococcaceae, Fusicatenibacter, Bacteroides, Veillonella*, and *Eubacterium hallii* (Crobach et al., 2020).

## Infant Gut Microbiota Characteristics and Possible Explanations for the Low Pathogenicity of *C. difficile* in Neonates

During childhood, the intestinal microbiota is subject to many factors that shape the microbiota on short- and long-term. Apart from the maternal-fetal transmission of certain bacterial components, the microbiota is influenced by the type and the time of birth, the place of birth (hospitals or home births), the type of feeding (breastfeeding or artificial feeding), administration of probiotic and prebiotic supplements, dietary factors, antibiotics and other drugs, sex, and other genetic differences and environmental factors, such as exposure to pets, number of family members, rural or urban environment, hygiene, geographical factors (Combellick et al., 2018; Mohammadkhah et al., 2018; Akagawa et al., 2019).

Taking into account the numerous factors that could influence the intestinal microbiota of the newborns, the healthy profile of this age group is considered to be represented by the types of gut bacteria and the abundances found in vaginally delivered, exclusively breastfed and not exposed to antibiotics neonates (Arboleya et al., 2015; Combellick et al., 2018; Mohammadkhah et al., 2018; Akagawa et al., 2019). It has been hypothesized that bacterial colonization of the digestive tract begins *in utero*, the healthy placenta bearing a low biomass microbiome, composed of non-pathogenic species belonging to the *Tenericutes, Firmicutes, Bacteroidetes, Proteobacteria*, and *Fusobacteria* phyla, while in the amniotic fluid predominate *Proteobacteria*. However, the results reported by different studies are contradictory and depend on the sampling method and the culture-based or molecular-based approaches. Recent metagenomic studies conclude that the placenta does not harbor a specific, consistent and functional microbiota (Gschwind et al., 2020). The meconium harbors a cultivable microbiota, initially dominated by *Bacteroides-Prevotella*. The digestive contamination of the fetus occurs most probably by swallowing the amniotic fluid, starting with the 10th week after conception (Chong et al., 2018). The transfer of *Enterococcus faecium* from pregnant female mice into meconium was also demonstrated experimentally (Jimenez et al., 2008). All these lead to the conclusion that changes in the maternal internal environment may affect both fetal and newborn development (Zhuang et al., 2019).

### *C. difficile* Colonization in Infants

Neonates are uniquely susceptible to *C. difficile* colonization because of the neonatal intestine’s immaturity and intestinal microbiota instability (Lees et al., 2016). The main source of colonization seems to be the environmental exposure to *C. difficile* spores within the nursery or healthcare environment rather than the mother, the rates of *C. difficile* detection increasing with the length of stay in these units.

In infants <1 month of age, *C. difficile* has an average colonization rate of 37%, ranging between 0 and 61%. Between 1 and 6 months of age, the colonization rate is still high at 30% and drops to about 10% by the end of the first year of life (Kuiper et al., 2017). However, the colonization rates reported by different studies vary from 14 to 71% in children <12 months of age. This age group is most commonly colonized with non-toxigenic strains and they are asymptomatic (Khalaf et al., 2012). The asymptomatic carriage rate continues to drop until about 3 years of age, when it stabilizes to carriage rates of 0–3%, similar to those found in adults, together with a progressive raise in serum IgG antibody concentrations against toxins A and B between birth and 24 months of age (Antonara and Leber, 2016). Around 3 years of age, the intestinal microbiota of the child is stabilized, acquiring the characteristics of the adult microbiota, which might explain the increase of symptomatic CDI starting with this age.

Moreover, other studies are assuming that asymptomatic carriage of *C. difficile* is common in the young individuals of many other species, including dogs, pigs, and cattle (Deng and Swanson, 2015). In puppies, the association between lower bacterial community diversity and *C. difficile* colonization was statistically significant, and certain bacterial taxa were preferentially associated with *C. difficile* colonization (Berry et al., 2019). Similar associations have also been found in human studies. Unweaned puppies that were not colonized with *C. difficile* had higher relative abundance of taxa from the clostridia genera than unweaned puppies that were colonized with *C. difficile* (Berry et al., 2019).

Many studies have found higher colonization rates with *C. difficile* in formula-fed infants than in breastfed infants (Cooperstock et al., 1983; Jangi and Lamont, 2010). Also, the breastfed infants colonized by *C. difficile* had significantly lower colony counts than formula-fed infants, probably because the human colostrum contains neutralizing antibodies to toxins A and B (Rolfe and Song, 1995; Jangi and Lamont, 2010).

There are no studies comparing *C. difficile* carriage rate regarding the delivery mode, but it might have persistent effects on microbiota beyond infancy. The lack of “bacterial baptism” of vaginal birth or other confounding factors associated with cesarean delivery, as well as maternal obesity, antibiotic administration, gestational age and breastfeeding pattern, could influence the *C. difficile* carriage rate (Kyne et al., 2000; Liu et al., 2019). However, there were found a significantly higher number of Clostridia in the stool of children vaginally delivered (VD) than in those delivered by C-section (CS) (Reyman et al., 2019). No association was found with prematurity as a risk factor for *C. difficile* infection (Lees et al., 2016).

The question arises whether the newborns and adults, which are asymptomatic carriers of *C. difficile*, might have a particular gut microbiota composition that allows colonization to occur without any clinical manifestations. Lack of disease has also been related to immature or diminished receptor sites for toxin A along the intestinal epithelium (Keel and Songer, 2007). In exchange, CDI may be more likely to manifest in certain populations of infants harboring pathological intestinal conditions, such as those with Hirschsprung’s disease, all these demonstrating the link between *C. difficile* colonization and intestinal homeostasis (Sammons et al., 2013).

In conclusion, the high carriage rate of *C. difficile* colonization in neonates can be explained by the immaturity of the neonatal intestine and the presence of a less complex intestinal microbiota, as compared to adults. However, postnatal microbial species, together with the lack of receptors for clostridial toxins protect help babies from the deleterious effects of *C. difficile* toxins.

### Neonate Gut Microbiota Signatures Associated With *C. difficile* Colonization

Considering that *C. difficile* only occasionally produces clinical manifestations in infants, one can state that a specific microbiota composition probably consolidating a specific environment in newborns helps protect babies from the deleterious effects of *C. difficile* toxins, which occur in dysbiotic adults.

The newborn microbiota is dominated by Gram-positive cocci, *Enterobacteriaceae* or *Bifidobacteriaceae*, with a sequential transition to a microbiota dominated by *Bifidobacteriaceae*. It is well known that *Bifidobacteria* upregulate IL-10 production by intestinal dendritic cells explaining the lack of clinical symptoms in infants colonized with *C. difficile* (Huurre et al., 2008). In CS neonates, decreased levels of T cells and CD4+ helper T cells were noticed, probably due to the failure of the immature infant immune system to activate an inflammatory response (Jangi and Lamont, 2010; Collins and Auchtung, 2017; Francino, 2018).

Prematurity might not be a risk factor for *C. difficile* infection (Lees et al., 2016), probably due to the fact that in general, the intestinal microbiota of the premature child is dominated by *Proteobacteria*, even if breastfed, and the species of *Clostridium* and *Veillonella* appear later. However, the microbiota of the premature infant is strongly influenced by pre- and postnatal antibiotic therapy (Staude et al., 2018). Hospitalization and antibiotic exposure induce indigenous microbiota imbalance (McFarland et al., 2016). Antibiotic treatment in neonate’s intensive care units (NICU) was associated with a lower *C. difficile* colonization rate, but colonization with *C. difficile* occurred rapidly after cessation of antibiotics. In children in the NICU, born prematurely, the colonization with *Bifidobacteriaceae* is delayed.

Breast milk protects against infections in infants due to the presence of immunological factors such as immunoglobulin A (IgA), including neutralizing antibodies to *C. difficile* toxins A and B (Jangi and Lamont, 2010). *Ruminococcus* (which is more commonly found in the gut of breastfed infants) is thought to inhibit the growth of *Clostridia*, thereby preventing colonization by *C. difficile* (Morelli, 2008).

Increased levels of immunoglobulin-producing cells in peripheral blood have been observed in CS infants, probably due to excessive exposure to antigens at the level of the vulnerable intestinal barrier. In addition, breastfeeding contributes to the maturation of the infant’s immune system and modulates microbiota development. The microbiota of breastfed children is less diverse but contains more *Bifidobacterium spp*., also explaining the protection against deleterious pro-inflammatory responses triggered by CDI (Derrien et al., 2019). However, *Bifidobacteria* significantly decrease in abundance upon cessation of breastfeeding.

In the clinical cases of neonate necrotizing enterocolitis (NEC), a decrease of bacterial diversity and of *Bacteroidetes* and *Firmicutes* phyla, as well as of *Bifidobacteria*, were observed, with the more frequent presence of potentially pathogenic organisms, such as *Staphylococcus aureus, Enterococcus*, *Escherichia coli, Shigella spp., Citrobacter spp., Klebsiella spp.* (Dobbler et al., 2017). Among the strictly anaerobic bacteria that have been associated with NEC, the majority belong to the *Clostridium* genus (e.g., *C. butyricum, C. neonatale, C. perfringens, C. paraputrificum, and C. difficile* have been associated with NEC in preterm neonates) (Zhou et al., 2015; Rozé et al., 2017; Schönherr-Hellec et al., 2018; Schönherr-Hellec and Aires, 2019). Moreover, a NEC-associated microbiota, such as *C. perfringens* has been identified in meconium samples (Heida et al., 2016).

## Manipulation of Gut Microbiota as Adjunctive Therapy of *C. difficile* Infection

The first step in *C. difficile* treatment is the de-escalation of antibiotic treatment. Depending on the degree of *C. difficile* risk induction, the antibiotics were divided into three groups: high (fluoroquinolones, 2nd and 3rd generation cephalosporins, clindamycin, ampicillin, broad-spectrum penicillins with inhibitors, except for ticarcillin with clavulanate, and piperacillin with tazobactam), moderate (macrolides, trimethoprim/sulfamethoxazole, other penicillins, and sulfonamides) and low risk (aminoglycosides, bacitracin, carbapenems, chloramphenicol, daptomycin, metronidazole, rifampicin, teicoplanin, tigecycline, tetracycline, and vancomycin) (Kukla et al., 2020). Current standard treatment for CDI involves treatment with antibiotics such as metronidazole, vancomycin, or fidaxomicin (Mills et al., 2018; Gnocchi et al., 2020). Vancomycin is the first-line antibiotic therapy for both first episode of infection and fulminant infections in adults (Esposito et al., 2015; Wang et al., 2020). Unfortunately, vancomycin is a strong disruptor of gut microbiota, while the rate of CDI recurrence after treatment cessation occurs in 20–30% of patients (Lessa et al., 2015; Winston and Theriot, 2016). On the other hand, metronidazole is used especially in the first episodes of mild acute CDI and less for severe disease because the concentrations in the colon become readily undetectable due to the fact that it is absorbed very quickly (Gnocchi et al., 2020). The rapid absorption from the gut is also reflected in a negligible effect on normal microbiota (Lessa et al., 2015; Chilton et al., 2018). Fidaxomicin can be used in *C. difficile* non-severe and also severe infections treatment due to the fact that it is poorly absorbed at the intestinal level, ensuring the persistence of killing concentration in the gut and has a narrow antimicrobial spectrum (e.g., Gram-positive and Gram-negative anaerobes and facultative aerobes) unaffecting the equilibrium of the normal intestinal microbiota (Tannock et al., 2010; Louie et al., 2011, 2012; Gnocchi et al., 2020). Moreover, in another study, vancomycin and metronidazole treatment, but not fidaxomicin were associated with the potentially pathogenic fungal operational taxonomic units’ emergence as well as with bacterial functions enriched for xenobiotic metabolism that could contribute to dysbiosis that could favor the occurrence, persistence and recurrence of CDI (Lamendella et al., 2018).

**FIGURE 1:**
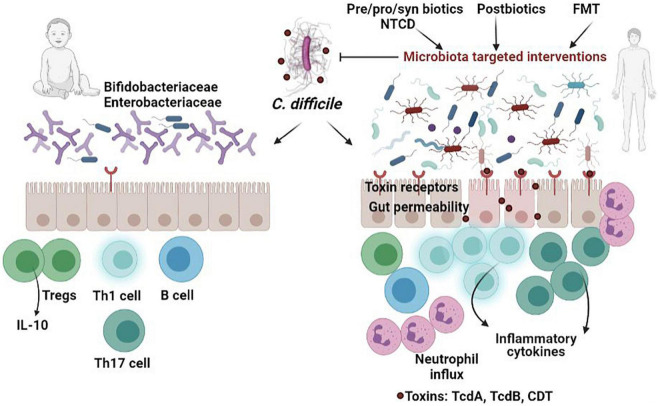
Comparison of *C. difficile* infection in neonates versus adults. The presence of postnatal microbial species (*Bifidobacteria* and *Enterobacteriaceae*) and immaturity of the immune system as well as the lack of receptors for clostridial toxins helps to protect babies from the deleterious effects of *C. difficile* toxins, which occur in dysbiotic adults. In individuals with disrupted microbiota, ingested *C. difficile* spores germinate in the presence of bile salts in the small intestine and target the colon epithelial cells, releasing the inflammatory toxins, which subsequently induce disruption of tight junctions, and production of inflammatory cytokines. Targeting the microbiota using various strategies-probiotics, prebiotics, synbiotics, postbiotics, non-toxigenic *C. difficile* (NTCD), fecal transplant (FMT)-has proven to be effective in alleviating CDI.

Adjunctive therapies are frequently used due to the important role of gut microbiota disturbances in *C. difficile* pathogenesis (Table 1 and Figure 1). Thus, specific manipulation of the microbiota to ameliorate dysbiotic changes and restore intestinal microbiota homeostasis could represent an essential part of the therapy (Table 2).

**TABLE 2:** Microbiota-centered therapeutic approaches with proven beneficial effects in CDI.

Type of microbiota-targeted intervention	Administration methods	Effects	References
**Probiotics/prebiotics/synbiotics**			
*Lactobacillus plantarum Inducia*	Use of xylitol as symbiotic to enhance the probiotic engraftment and effects	Total inhibition of *C. difficile* spores germination *in vitro*; reduction of mouse mortality	Ratsep et al., 2017
*Bifidobacterium longum Bifidobacterium breve*	Use oligo-fructosaccharides as a carbon source (symbiotic effect)	Reduction in toxicity	Valdes-Varela et al., 2016
*Bacillus clausii O/C*	Administered alone	Neutralization of *C. difficile* toxin inhibition of *C. difficile* toxins by bacterial secreted compounds (serine protease, M-protease)	Ripert et al., 2016; Aktories et al., 2017
*Bacillus thuringiensis*	Administered alone	Production of bacteriocin direct inhibition	Mills et al., 2018
*Enterococcus durans*	Administered alone and with bacteriocins (reuterin, nisin)	Production of durancin	Hanchi et al., 2017
*Lactobacillus reuteri*	Administered alone	Direct inhibition production of antibacterial substances such as reuterin obtained through fermentation of glycerol	Spinler et al., 2017
Multi-strain capsule (*Lactobacillus acidophilus NCFM, ATCC 700396; Lactobacillus paracasei Lpc-37, ATCC SD5275; Bifidobacterium lactis Bi-07, ATCC SC5220; B. lactis Bl-04, ATCC SD5219*)	Administered as multi-strain capsule	Probiotic adjunct therapy was associated with a significant improvement in diarrhea outcomes	Barker et al., 2017
Spores of *Firmicutes* phylum (e.g., SER-109; SER-262)	Administration of purified spores	Repopulation the gut microbiota	Khanna et al., 2016; Gnocchi et al., 2020
**Postbiotics**			
Filtered fecal supernatant	Administration of microbe-free fecal filtrates	Rapid shifts in gut microbial composition	Kelly et al., 2016; Hota et al., 2017; Ott et al., 2017
**Competition for resources**			
Non-toxigenic *C. difficile* (NTCD)			Gerding et al., 2015
**Fecal microbiota transplantation**			
Consortia of fecal bacteria quality-controlled and semi-standardized (e.g., RBX2660)	Use of fecal derivatives for the treatment of CDI	Repopulation of the gut microbiota	Orenstein et al., 2016

One way to modulate microbiota is using “biotics” with beneficial impact on resident microbiota that confer health benefits for the host, such as probiotics (e.g., *Saccharomyces boulardii, Lactobacillus*, *Bifidobacterium*, and probiotic mixtures), prebiotics, symbiotics or postbiotics (Surawicz et al., 2000; Na and Kelly, 2011; Maziade et al., 2015; Collins and Auchtung, 2017). According to International Scientific Association for Probiotics and Prebiotics (ISAPP)^1^, prebiotics are substrates different from fibers, that are selectively metabolized by host microorganisms. Synbiotics are complementary/synergistic mixtures comprising live microorganisms (probiotics) and prebiotics. In case of synergistics synbiotics the two components taken individually do not have to meet criteria for prebiotic or probiotic. Postbiotics are cellular fractions or structures prepared from inactivated microbes. The postbiotic preparations exclude filtrates or live cultures individual components, while inactivated probiotics are not considered automatically postbiotics, unless a health benefit is demonstrated.

Most data regarding the protective role of commensal bacteria against *C. difficile* infection were obtained by studying the effects of different probiotics. For example, *Bifidobacterium breve* (YH68), widely used in the field of food fermentation and biomedicine, has shown antibacterial activity against *C. difficile*, by inhibiting the growth, spore production, toxigenesis and virulence gene expression (Valdes-Varela et al., 2016; Yang and Yang, 2019), potentiating the effect of anti-*C. difficile* antibiotics *in vitro* (Yang and Yang, 2018) or preventing the occurrence of clinical manifestations *in vivo* (Yun et al., 2017). Probiotic use has been shown to decrease CDI incidence in high-risk populations by as much as 50%, especially when they are combined with prebiotics (Shen et al., 2017).

It was demonstrated that the administration of non-living bacteria or microbial components (e.g., proteins, lipids, or nucleic acids) has an immunostimulatory effect proving that the beneficial impact on the host health is due to the physical interaction of specific microbial components, but in order to be effective for a long period, these need continuous administration.

Many bacterial strains such as *Bacillus clausii* and *Lactobacillus reuteri* have been shown to secrete soluble compounds that directly inhibit *C. difficile* (Khalaf et al., 2012; Deng and Swanson, 2015; Antonara and Leber, 2016). Organisms that produce secondary bile acids, such as *Clostridium scindens*, enhance *C. difficile* colonization resistance (Winston and Theriot, 2016).

Fecal microbiota transplantation (FMT) is considered the most effective microbiota-targeted intervention for the treatment of antibiotic-refractory CDI (Arbel et al., 2017), but however, the long-term effects, including the risk of other diseases, are not known (Schaffler and Breitruck, 2018).

Another therapeutic approach is the administration of non-toxigenic *C. difficile* strains or a mixture of spore-forming commensals, which act by providing nutritional niche competition. Despite their efficiency in decreasing CDI recurrence, there is the risk of switching to the toxigenic phenotype (Brouwer et al., 2013; Khanna et al., 2016).

## Conclusion

The available studies suggest that *C. difficile* colonization and infection are influenced by the presence, absence or abundance of certain bacteria in the human gut, which could generate favorable conditions for germination, proliferation and production of clostridial toxins which, on their turn, will alter the integrity of the intestinal mucosa. Therefore, the clinical manifestations and severity of CDI are linked to gut dysbiosis, that could have multiple causes, among which the administration of high-risk antibiotics. The presence of protective microbial species, together with the particularities of the immune system and lack of receptors for clostridial toxins could explain the fact that in children, despite the high carriage rate, the symptomatic and severe cases are rare. However, if there is a gut microbiota composition predisposing to *C. difficile* asymptomatic carriage or clinical infection still needs clarification. Also, the mechanisms involved in *C. difficile* crosstalk with the commensal microbiota and/or particular soluble compounds remain only partially explained. Adjunctive microbiota-targeting therapies based on probiotics, prebiotics, postbiotics, synbiotics, non-toxigenic bacteria or fecal microbiota transplantation proved to be very useful for the therapeutic management of CDI.

## Author Contributions

All authors contributed equally to this manuscript with the conception and design of the study, literature review and analysis, drafting and critical revision and editing, and final approval of the final version.

## Conflict of Interest

The authors declare that the research was conducted in the absence of any commercial or financial relationships that could be construed as a potential conflict of interest.

## Publisher’s Note

All claims expressed in this article are solely those of the authors and do not necessarily represent those of their affiliated organizations, or those of the publisher, the editors and the reviewers. Any product that may be evaluated in this article, or claim that may be made by its manufacturer, is not guaranteed or endorsed by the publisher.

## Footnotes

1
https://isappscience.org/a-roundup-of-the-isapp-consensus-definitions-probiotics-prebiotics-synbiotics-postbiotics-and-fermented-foods/
